# HIV blocking antibodies following immunisation with chimaeric peptides coding a short N-terminal sequence of the CCR5 receptor

**DOI:** 10.1016/j.vaccine.2008.08.025

**Published:** 2008-10-23

**Authors:** Benjamin M. Chain, Mahdad Noursadeghi, Michelle Gardener, Jhen Tsang, Edward Wright

**Affiliations:** Division of Infection and Immunity, Windeyer Building, UCL, 46 Cleveland St., London W1T 4JF, UK

**Keywords:** CCR5, Antibody, Chimaeric peptide, Blocking antibodies

## Abstract

The chemokine receptor CCR5 is required for cellular entry by many strains of HIV, and provides a potential target for molecules, including antibodies, designed to block HIV transmission. This study investigates a novel approach to stimulate antibodies to CCR5. Rabbits were immunised with chimaeric peptides which encode a short fragment of the N-terminal sequence of CCR5, as well as an unrelated T cell epitope from Tetanus toxoid. Immunisation with these chimaeric peptides generates a strong antibody response which is highly focused on the N-terminal CCR5 sequence. The antibody to the chimaeric peptide containing an N-terminal methionine also recognises the full length CCR5 receptor on the cell surface, albeit at higher concentrations. Further comparison of binding to intact CCR5 with binding to CCR5 peptide suggest that the receptor specific antibody generated represents a very small fragment of the total anti-peptide antibody. These findings are consistent with the hypothesis that the N-terminal peptide in the context of the intact receptor has a different structure to that of the synthetic peptide. Finally, the antibody was able to block HIV infection of macrophages in vitro. Thus results of this study suggest that N-terminal fragments of CCR5 may provide potential immunogens with which to generate blocking antibodies to this receptor, while avoiding the dangers of including T cell auto-epitopes.

## 1 Introduction

Classical vaccination strategies applied to HIV infection have proved to be ineffective. A significant proportion of antibody against the virus is non-neutralising, and indeed some antibody may enhance viral infectivity [1,2] The majority of neutralising antibody is directed against the gp120 glycoprotein on the viral surface. This protein shows extensive variability, and most antibodies are strain specific. Some cross-strain neutralising antibodies have been reported [3,4] and efforts are in progress to focus the immune response on the epitopes recognised by these antibodies. However, alternative strategies to inhibit HIV are also of interest.

An alternative target for HIV blocking antibodies are the receptors used by virus to gain entry into the cell. The CD4 molecules plays a key role in T cell function, and antibodies against it are likely to be pathogenic. However, the CCR5 chemokine receptor offers a more attractive target. This receptor is absent in approximately 1% of the Caucasian population [5], and these individuals show no gross symptoms of immunodeficiency, although recent reports have suggested a possible role for CCR5 in mycobacterial responses [6] and West Nile virus infection [7]. CCR5 deficiency is associated with almost complete protection against HIV infection [8,9] and even CCR5 heterozygous individuals, which show haplotype insufficiency, show a slower progression to AIDS [10]. Genetically determined overexpression of CCR5 ligand CCL3L1 is also associated with partial protection [11]. Finally, small molecular weight CCR5 antagonists can block HIV entry, and one such inhibitor is in clinical use [12]. Thus, CCR5 levels are quantitatively and qualitatively a key parameter in determining the course of HIV infection, and the subsequent development of AIDS.

Several groups have recently investigated the possibility of raising antibodies against CCR5 [13–17], and have used recombinant proteins, recombinant viruses or synthetic cyclic peptides to provide proof of principal evidence that the strategy can work. The safety of autoantigen driven vaccine strategies remains a cause for concern, however. A trial of therapeutic vaccination in Alzheimer patients using the amyloid fragment Aβ, was discontinued because of adverse side effects attributed to the autoimmune response [18], although the damage may have been due to autoimmune cellular rather than humoral responses. Cellular autoimmune responses against the CCR5 receptor are likely to be pathogenic, since they may lead to elimination of dendritic cells, macrophages, T cells and any other cell types which express this receptor. In this study, therefore, we explore the possibility of raising an immune response to the CCR5 receptor, using a very short N-terminal fragment of the receptor, coupled to a well characterised epitope of tetanus toxoid [19,20]. Since the immunogen contained seven amino acids of CCR5 sequence, the possibility of including a CD4 or CD8 T cell auto-epitope is avoided. The N-terminal sequence was chosen, since it may adopt a looser configuration than the other extracellular loops.

Our study shows that this chimaeric peptide can indeed stimulate antibody which recognises intact receptor, and that this antibody can reduce HIV infectivity in vitro. However, only a small proportion of the anti-peptide antibody crossreacts with receptor, suggesting that the N-terminal region of the native CCR5 receptor may be structurally distinct from the synthetic peptide immunogen.

## 2 Methods

### 2.1 Peptides

The sequence of the peptides used in this study are given below: All peptides were synthesised by the Protein and Peptide Chemistry Department at Cancer Research UK. Purity was greater than 80% as measured by HPLC. Peptide CCR5 was also supplied coupled to Keyhole Limpet Haemocyanin (KLH) via an additional terminal cysteine, using the protocol described below for bovine serum albumin (BSA).

### 2.2 Cell lines

U87 cells (a human glioma derived line) expressing CD4 and CCR5 (U87-CD4-CCR5) were obtained from Drs. Littman and Deng, via the Centralised Facility for AIDS Reagents (Repository Reference ARP069 and ARP072) (http://www.nibsc.ac.uk/spotlight/aidsreagent/) supported by EU Programme AVA/MRC (contract QLKZ-CT-1999-00609) and the UK Medical Research Council. The cells were maintained in selection medium (15% foetal calf serum, DMEM) containing G418 (300 μg/ml) and puromycin (1 μg/ml, only for CCR5 expressing cells) as described in detail in the data sheet for these cells lines.

The human glioma cell line NP2, expressing CD4 and CCR5 or CXCR4 [21], were maintained at 37 °C and 5% CO_2_ in 10% FCS, DMEM.

Macrophages were prepared by culture of monocytes isolated from healthy volunteers in human serum and M-CSF as described previously [22]. The study was approved by the joint UCL/UCLH NHS Trust Human Research Ethics Committee and written informed consent was obtained from all participants. This protocol yields macrophages at >95% purity by morphology and CD14 staining.

### 2.3 Immunisations

Two sets of immunisations were carried out at the Biological Services Unit, UCL.

#### 2.3.1 Experiment 1

Three rabbits were immunised with each peptide CCR5.1 and CCR5.2. Primary immunisations were carried out with peptide (total 1 mg) emulsified in complete Freund's adjuvant (CFA, Sigma), at four subcutaneous sites. The rabbits were boosted at day 15, 22, 29 and 42 with peptide emulsified in incomplete Freund's adjuvant (IFA). All rabbits were sacrificed and exsanguinated by cardiac puncture on day 63. Serum (preimmune, test and final) were collected using standard methods, and stored at −20 °C until needed.

#### 2.3.2 Experiment 2

Three rabbits were immunised with peptide CCR5.5 conjugated to KLH. Primary immunisations were carried out with conjugate (Total 0.4 mg) emulsified with CFA, at four subcutaneous sites. Rabbits were boosted on days 14, 21, 28 and 44 with KLH/peptide conjugate (Total 0.2 mg) emulsified with IFA. A further three rabbits were immunised with peptide CCR5.5 bound to magnetic streptavidin beads (M-PVA SAV1, Chemagen). Beads (25 mg) were washed in PBS, and mixed with peptide CCR5.5 (100 μg). After 15 min, the suspension of beads was mixed with adjuvant (Imject^®^ ALUM, Pierce), and shaken vigorously to produce an emulsion. Immunisation schedule was as for CCR5.5/KLH, except that the same bead/peptide suspension was used for all immunisations. A total of 100 μg peptide was injected at each immunisation. All rabbits were sacrificed and exsanguinated by cardiac puncture on day 63. Serum (preimmune, test and final) were collected using standard methods, and stored at −20 °C until needed.

### 2.4 Conjugation of peptides to BSA

CCR5.3 and CCR5.4 were conjugated to BSA using sulphosuccinimimidyl 4-(*N*-maleimidomethyl) cyclohexane (Sulpho-SMCC), Sigma–Aldrich, Poole, Dorset). Briefly, 0.6 μmol BSA were reacted with 17 μmol Sulpho-SMCC in phosphate buffered saline (PBS) for 1 h. The BSA/SMCC conjugates were exchanged into sodium phosphate buffer (0.1 M, pH 6) using PD-10 desalting column (Amersham Biosciences). Meanwhile, 10 μmol of each peptide were dissolved in 1 ml borate buffer (0.1 M, pH 8) and reduced by the addition of sodium borohydride (130 μmol); excess borohydride was destroyed by brief acidification. Each reduced peptide solution was mixed with half the BSA/SMCC, and incubated overnight at 4 °C. The final conjugate was dialysed against PBS and stored frozen in aliquots.

### 2.5 Conjugation of BSA/peptide to Sepharose

Fifteen to 20 mg of each peptide/BSA conjugate was coupled to CNBr-activated Sepharose 4B (Sigma–Aldrich) using the manufacturer's instructions. The coupled matrix was extensively washed in high (pH 8) and low (pH 4) pH buffers, and stored in PBS/azide until used for affinity purification.

### 2.6 Antibody purification

CCR5.3/BSA/Sepharose (approximately 1 ml) was washed with 20 ml PBS. 5–10 ml serum from immunised rabbits was mixed with the Sepharose and incubated for 20 min to allow binding to the immunoadsorbent. The flow through was then collected, and the column was washed with a further 20 ml PBS. Bound antibody was eluted with 5 ml glycine solution (0.1 M, pH 2.5), and 400 μl fractions collected. 6 μl Tris solution (1 M) was added to each collecting tube to bring the pH back to neutral as rapidly as possible. Each fraction was tested for CCR5.3 binding activity by ELISA, and fractions containing the highest titres (typically fractions 4–8) were pooled, and filter sterilised. For functional assays, the purified antibody was exchanged into complete culture medium using PD-10 columns, following manufacturer's instructions.

### 2.7 Antibody radiolabelling

Forty micrograms purifed antibody was mixed with 0.2 mCi NaI^125^ (Amersham) and 10 μg chloramine T (Sigma) and incubated for 60 s. The reaction was terminated by adding sodium metabisulphite (10 mg), and excess I^125^ quenched by addition of potassium iodide (10 mg). Labeled protein was purified from reactants by column chromatography on a PD10 column (Amersham Biosciences) according to manufacturers’ instructions, and stored in PBS at 4 °C until used. Specific activity was about 1.2 × 10^6^ cpm per μg protein.

### 2.8 ELISA

96 well ELISA plates (Nunc) were coated with BSA/peptide conjugates (05–1 μg/ml) in coating buffer (0.1 M sodium bicarbonate, pH 8.5), overnight at 4 °C. All wells were washed (washing buffer PBS 0.1% Tween-20) and blocked by addition of 2% low fat milk (Tesco) in PBS for at least 2 h at room temperature.

Sera or antibodies were diluted in washing buffer and added at the concentrations shown, for 1.5–3 h at room temperature. Bound antibody was detected using alkaline phosphatase coupled sheep anti-rabbit (Sigma) antibody (1:2000 dilution) and substrate (PNPP FAST, Sigma). Results are shown either as optical density (405 nm) or as log_3_ titre. The titre is calculated as the largest dilution factor which gives a reading above twice the mean background optical density (no first layer).

### 2.9 Flow cytometry

U87 transfected cells (5 × 10^4^) were incubated in 50 μl PBS containing 10% goat serum (Sigma) and 0.1% sodium azide (blocking buffer) for 30 min at 4 °C. Test or control sera at various dilutions (in blocking buffer) were added, and incubated for 2–3 h at 4 °C. Cells were washed and bound antibody detected using goat anti-rabbit FITC (R&D Systems). A positive control monoclonal rat anti-human CCR5 was obtained from Drs. J. McKeating and C. Shotton, via the Centralised Facility for AIDS Reagents (Repository Reference ARP3214.1) (http://www.nibsc.ac.uk/spotlight/aidsreagent/).

### 2.10 Functional assays of blocking activity

#### 2.10.1 Inhibition of HIV on transfected NP2 cells

5 × 10^3^ NP2 cells stably expressing CD4 and either CCR5 or CXCR4 were seeded into a 96-well plate 24 h prior to treatment with the CCR5 antagonist TAK779 [23] (10 μM, NIBSC, UK), purified CCR5 antibody or control antibody. The cells were incubated at 37 °C for 1 h after which the existing medium was replaced with fresh supplemented medium with antibody and 100 focus-forming units of virus. The HIV-1 strains SF162, BaL and the primary isolate SL2 were used in these experiments. After 2 h at 37 °C the cells were washed twice and medium containing antibody was placed back onto the cells for a further 48 h. The cells were then fixed in ice cold acetone–methanol (1:1) and stained in situ for p24 expression as described previously [24]. The blocking experiments with NP2 cells were also repeated using the protocol described below for macrophages, with very similar results to those obtained with the initial protocol.

#### 2.10.2 Inhibition of HIV on primary human macrophages

Macrophage cultures were pre-incubated with affinity purified rabbit anti-CCR5 immunoglobulin or pre-immune rabbit IgG for 1 h at room temperature and then inoculated with HIV-1 BaL (passaged on primary human macrophages and titred on NP2 cells) using doubling dilutions of virus prepared in medium with anti-CCR5 or control antibody. The inoculum was removed after overnight incubation and the intracellular p24 staining assessed 3–4 days later as described [24].

## 3 Results

**Fig. 1:**
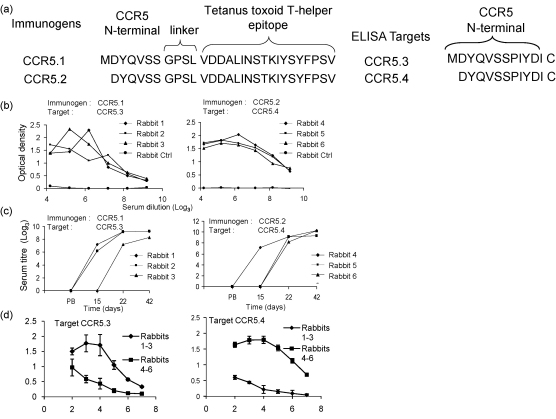
Anti-peptide antibody responses in rabbits immunised with peptides CCR5.1 and CCR5.2. (a) The sequences of peptides used for immunisation and ELISA, showing the sequences derived from CCR5, the linker sequence and the Tetanus toxoid T cell epitope sequence. (b) Antisera from the final bleed from rabbits immunised with CCR5.1 (left panel) or CCR5.2 (right panel) were diluted 1:100 in blocking buffer, and then diluted over a range of threefold dilutions as shown. Antibody reactivity was tested by ELISA against peptides CCR5.3 (left panel) and CCR5.4 (right panel) as described in Section 2.8. Each point shows the mean of duplicate wells. Each rabbit serum was tested at least three times. (c) Antibody titre from prebleed (PB) or test bleeds collected at different times postimmunisation as shown. Each point shows the mean of duplicate wells. Titres of 100 or less are shown as zero. (d) Cross reactivity of antisera to heterologous peptides. Rabbits 1–3 were immunised with peptide CCR5.1 (with N-terminal methionine) while rabbits 4–6 were immunised with CCR5.2 (without N-terminal methionine). Each serum (final bleed) was then tested against the homologous and heterologous peptide as shown. Each point shows mean of three sera with standard error of the mean.

CCR5.1 peptide incorporates the first seven amino acids of the full length sequence of human CCR5, followed by a linker/spacer and an MHC class II promiscuous T cell epitope from tetanus toxoid [19,20]. Remarkably, there is no information in the literature about whether the methionine at position 1 is still present or is cleaved before CCR5 is exported to the cell membrane. A second peptide, in which the methionine was absent was therefore tested in parallel. Sera were first tested by ELISA, using as target antigen peptides CCR5.3 and CCR5.4, which contain the first twelve amino acids of the CCR5 receptor (Fig. 1), with (CCR5.3) or without (CCR5.4) the initial methionine. Each peptide was conjugate via an additional C-terminal cysteine to BSA. A representative ELISA for all six sera showed that all rabbits gave strong antibody responses to peptides containing the CCR5 sequences to which they had been immunised (Fig. 1), with titres of greater than 20,000 (9, log_3_). Titres rose rapidly after 1 or 2 boosts (Fig. 1b), and appear to be reaching a plateau by the time the rabbits are sacrificed. The sera from the rabbits immunised with methionine-containing peptide preferentially recognised the target peptide with an N-terminal methionine (Fig. 1c, left), while the sera from rabbits immunised with peptide without methionine preferentially recognised peptides without methionine (Fig. 1c, right).

**Fig. 2:**
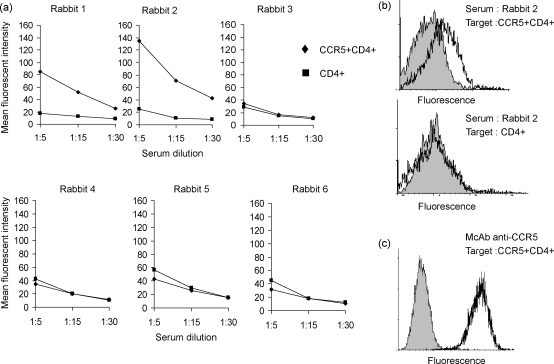
Anti-CCR5 antibody responses in rabbits immunised with peptides CCR5.1 and CCR5.2. Final bleed antisera from rabbits 1–6 were tested for binding to CCR5+/CD4+ U87 cells (diamonds) or control CD4+ U87 cells (squares) by flow cytometry. (a) Mean fluorescent intensity at various dilutions for each rabbit. This experiment has been repeated three times. (b) A representative histogram for prebleed (filled histogram) or final bleed (solid line) for rabbit two serum, tested against CCR5+/CD4+ U87 cells (top panel) or control CD4+ U87 cells. Each serum was tested by flow cytometry at least four times. (c) Control staining of a monoclonal anti-CCR5 (solid line) from the Centralized AIDS Repository or isotype control (filled histogram).

The sera were then tested for binding to CCR5 by flow cytometry, using CCR5 transfectants as targets. A summary showing mean fluorescence intensities at a variety of concentration for all six rabbits is shown in Fig. 2a, and a representative fluorescence histogram for one rabbit (rabbit 2) is shown in Fig. 2b. Two of the three sera recognising the methionine-containing N-terminal peptide bound CCR5 transfected cells, but not to controls. In contrast, none of the sera from the three rabbits immunised with the truncated peptide bound to CCR5. All further experiments used the methionine-containing sequence.

**Fig. 3:**
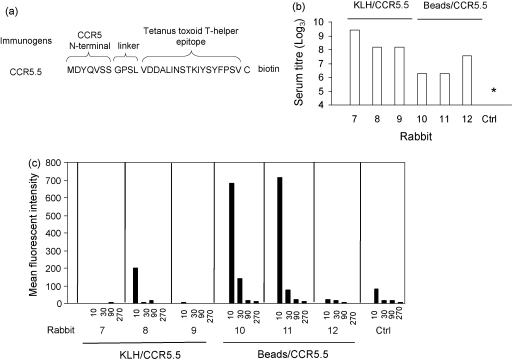
Antibody responses in rabbits immunised with peptide CCR5.5. (a) Sequence of CCR5.5. (b) Anti-peptide titre in final bleeds from rabbits 7–12, measured by ELISA using CCR5.3 peptide as target. Rabbits 7–9 were immunised with CCR5.5/KLH conjugate with CFA, rabbits 10–12 were immunised with CCR5.5 peptide coupled to magnetic beads with Alum. Ctrl shows the results using rabbit serum from an immunised rabbit. Asterisk shows titre <100. The figure shows one representative experiment, from at least three with each serum. (c) Final bleed antisera from rabbits 7–12 were tested for binding to CCR5/CD4+ U87 cells by flow cytometry. Each panel shows mean fluorescent intensity at dilutions of 1:10, 1:30, 1:90 and 1:270 for each rabbit. Ctrl shows the results using sera from an unimmunised rabbit. The figure shows one representative experiment, from at least three with each serum.

The immunogenicity of the peptides may have been limited by the presence of only a single T helper epitope in the immunising peptide, and/or by the fact that peptide immunogens are intrinsically univalent. Two additional sets of immunisation were therefore carried out with peptide CCR5.5, which is identical to CCR5.1, except for the addition of a C-terminal biotinylated cysteine. The C-terminal cysteine was used to couple to a strong “carrier protein”, KLH, and the conjugate emulsified in Freund's adjuvant was used to immunise rabbits 7–9. In parallel, the biotin moiety was used to couple to inert streptavidin-coated magnetic beads, and the beads were resuspended in Alum adjuvant and used to immunise rabbits 10–12. The titres of all sera at the time the rabbits were sacrificed tested against CCR5.3 is shown in Fig. 3. The titres were generally somewhat lower than those obtained with the first set of immunisations, with the lowest titres obtained following bead/Alum immunisation. However, in all cases the titres were at least 100-fold higher than control or preimmune sera, which gave titres of less than 4 (log_3_, 100). The six sera were further tested for binding to CCR5 receptor as above (Fig. 3b). None of the three sera from rabbits immunised with KLH conjugate showed significant binding to the transfectants. In contrast two of three rabbits immunised with CCR5.1 conjugated magnetic beads showed significant binding to CCR5 transfected cells (Fig. 3b), but not controls (not shown).

**Fig. 4:**
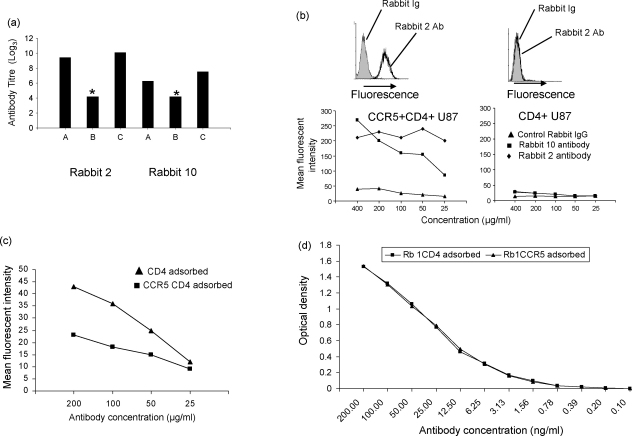
Purification of antibody from serum. (a) Anti-peptide binding activity of unpurified serum (A), flow through from affinity column of peptide CCR5.3 (B), and pooled eluate from affinity column (C) from rabbit 2 and rabbit 10. The titre of each sample was measured by ELISA using CCR5.3 as target. The purified antibody preparations were first adjusted to a concentration of 400 μg/ml. Asterisk indicate titre less than 100. (b) The purified antibody preparations, or an equal concentration of purified rabbit Ig were tested for binding to CCR5/CD4 expressing U87 cells or control CD4 expressing U87 cells by flow cytometry. Top two panels shows representative histograms of rabbit 2 purified antibody (80 μg/ml solid line) or control Ig (80 μg/ml, shaded) staining of CCR5+CD4+ U87 cells (left) or control CD4+ U87 cells (right). Bottom two panels show Mean Fluorescent Intensity at different concentrations of purified antibody from sera of rabbit 2 (diamonds), rabbit 10 (squares) or control rabbit Ig (triangles), tested either on CD4+CCR5+ expressing cells (left) or CD4+ expressing cells (right). (c) 100 μl purified antibody (40 μg protein) from rabbit 2 serum was adsorbed on 2 × 10^7^ CCR5+CD4+ U87 cells (squares) or 2 × 10^7^ CD4+ U87 cells (triangles) for 1 h at 4 °C. The cells were removed by centrifugation and the supernatant tested for binding by flow cytometry at different concentrations of protein. The figure shows results for one of two experiments. (d) The same two samples of adsorbed antibody as in (c) were tested for binding to peptide CCR5.3 by ELISA.

The results shown in Figs. 1–3 demonstrate a very large difference between the titre of anti-peptide antibody as measured by ELISA and as measured by binding to intact receptor by flow cytometry. To investigate this further, the antibody with anti-peptide activity was purified from serum of rabbit 2 (first set of immunisations) and rabbit 10 (second set of immunisations) by affinity chromatography, using peptide CCR5.3/BSA conjugate bound to Sepaharose 4B. The titre of antibody remained high after purification (Fig. 4a), although when adjusted for volume of purified antibody, recovery of active antibody was about 50% for both sera.

In order to test whether the anti-peptide antibody fraction contained the antibody which bound to the CCR5 receptor, the two purified antibody preparations were further tested for binding to CCR5 expressing transfectants (Fig. 4b). Both purified samples bound specifically to CCR5 transfectants, but not to control cells. In order to estimate the proportion of total peptide binding antibody which bound to CCR5, a purified antibody sample was absorbed on excess target CCR5 cells (2 × 10^7^) or on control cells not expressing CCR5, and then tested for binding to CCR5 (Fig. 4c) and to CCR5.3 peptide (Fig. 4d). Adsorption on receptor expressing cells removed the majority of receptor binding activity as expected (Fig. 4c), but did not significantly alter the peptide binding ability as measured by ELISA (Fig. 4d). Thus receptor binding activity can be attributed to a small proportion of anti-peptide binding antibody.

In order to obtain more quantitative figures for antibody concentration and antibody affinity, purified antibody was labeled to high specificity (approximately 1.5 × 10^6^ cpm/μg protein) by chloramine T iodination. Approximately 50% of this activity bound to CCR5.3/BSA/Sepharose beads, the remaining 50% representing either non-specific protein which copurified from the affinity column, or antibody damaged/destroyed by the iodination procedure. Specific binding to CCR5 transfectants was measured, either by comparing binding to control cells without CCR5, or by the addition of a large excess of unlabeled antibody. In three separate experiments, less than 0.2% of the activity bound to transfected cells, even under optimal conditions of cell numbers/antibody concentration. This low% binding is consistent with the binding data shown in Fig. 4c/d. The level of binding was too low to allow any accurate measurements of affinity/antibody concentration.

**Fig. 5:**
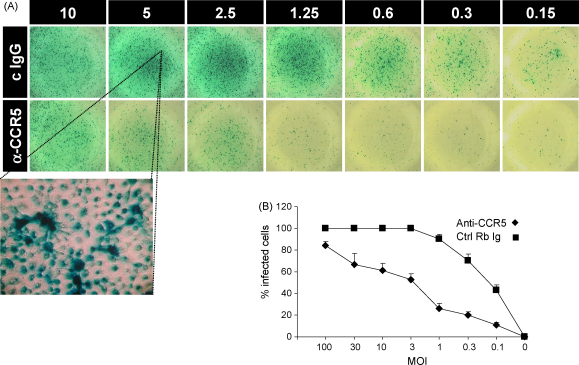
Inhibitory activity of anti-CCR5 antibodies on HIV infectivity. Human macrophage cultures were infected with HIV BaL at various MOIs in the presence of anti-CCR5 purified antibody (80 μg/ml) or control rabbit Ig. (A) Representative p24 staining after 4 days of culture. (B) Average % of p24 positive cells/well of triplicate cultures (mean and S.E.M., one experiment of three).

Although binding of antibody to CCR5 required large concentrations of antibody, we tested both unpurified sera and purified antibody in HIV neutralisation assays. Inhibition experiments using primary macrophage cultures as targets, showed strong inhibition of HIV BaL infection (Fig. 5). In contrast, experiments using several strains and NP2 CCR5 transfectants showed only 10–20% inhibition in infectivity compared to control (not shown). In comparison the CCR5 inhibitor TAK779 gave inhibition of around 50%, ranging between 20 and 70%.

## 4 Discussion

The primary objective of this study was to test the hypothesis that a short N-terminal fragment of the CCR5 would stimulate antibodies which recognise the intact receptor on the cell surface, and to characterise these antibodies. The results suggest that the basic hypothesis is correct, but suggest that the three-dimensional conformation of this domain may have structural constraints not previously appreciated.

The basic strategy uses a chimaeric immunogen containing a short N-terminal sequence of CCR5 to act as the B cell immunogen, and a C-terminal tetanus toxoid T helper cell epitope, separated by a four amino acid linker region. Immunisation with these chimaeric peptides successfully generated a strong antibody response to a peptide coding the N-terminal sequence of CCR5. The antibodies generated were highly focused on the N-terminal itself, since inclusion or removal of an additional methionine generated antisera with minimal crossreactivity.

Although numbers of animals tested were too small for statistical analysis, no antisera to the methionine-less peptide recognised the intact receptor, suggesting that this amino acid is retained at the cell surface. Although CCR5 protein has not been sequenced, these results are consistent with the known preference of methionine aminopeptidase, which cleaves methionine inefficiently where the adjacent amino acid has a large side chain (e.g. aspartic acid in this example) [25]. Similarly, a methionine at position 1 is retained in the three-dimensional structure of rhodopsin, a G protein coupled receptor homologue of CCR5 [26]. This observation raises some questions about recent attempts to determine or model structures for the CCR5 N-terminal peptide in complex with HIV gp120, since in these studies the N-terminal methionine is generally absent [27,28].

Three different immunogens were used in this study, the chimaeric peptide alone (with CFA), the chimaeric peptide coupled to KLH (with CFA), and the chimaeric peptide coupled to streptavidin-coated magnetic beads (with Alum). All three stimulated good titres of anti-peptide antigen, and there was no apparent advantage to incorporating additional T cell epitopes by coupling to KLH. Lower titres of anti-peptide antibody were obtained using Alum as adjuvant, consistent with the known weaker adjuvant activity of Alum compared to CFA. However, other factors, such as the concentration of peptide, and the influence of magnetic beads may also have played a role, and these different variables need to be analysed further.

Serum from four rabbits detected cell surface CCR5 by flow cytometry. The concentration of antibody required was much higher than that required to detect peptide by ELISA (microgram/ml versus nanogram/ml). We hypothesised that the antibody is made up of two populations with different specificities, one major species which recognises the peptide, but not the intact protein, and a minor species which recognises both. In support of this hypothesis, absorption with CCR5 expressing cells at least for antibody from one rabbit, decreases the titre of cell binding antibody significantly, but does not detectably change the titre of anti-peptide activity. Furthermore, absorption of radiolabeled antibody with CCR5 expressing cells only removes about 0.1–0.2% of the total labeled population, although at least 50% can still bind peptide-coupled sepharose beads. If correct, the hypothesis suggests that the N-terminal peptide of CCR5 may have a more well-ordered structure than previously supposed. An alternative explanation is that CCR5 is post-translationally modified on the cell surface. Indeed some tyrosine residues are known to be sulphated in intact CCR5, including tyrosine at position 3 [29].

Interestingly, serum from two of the three rabbits immunised with CCR5.5/beads/Alum reacted well with CCR5, despite showing lower total anti-peptide titres than those immunised with peptide/CFA. Further studies will be required to determine whether this is due to the presentation of the peptide on the surface or beads, or whether it reflects the different adjuvants used in each case.

We tested the ability of the antibodies to block HIV entry and replication, in both transfected cell lines, and primary human macrophage cultures. Unsurprisingly, the antibodies were poor inhibitors of HIV infectivity as measured on NP2 CCR5 transfectants, which had very high levels of receptor expression (not shown) and were difficult or impossible to saturate. In contrast, the antibodies showed a reasonably good level of inhibition when tested using primary macrophage targets. The reasons for this difference were not explored, but a plausible hypothesis is the extremely low level of CCR5 receptor expression on the primary macrophages compared to the transfected cell lines (not shown).

In conclusion, this study demonstrates that very short linear peptide epitopes, which cannot code for intact T cell auto-epitopes, may be used to generate an antibody response to the CCR5 protein. These results thus complement several other reports of stimulation of antibodies to CCR5 which block HIV entry/infection, using recombinant proteins or synthetic peptides as immunogens [13,14,16]. Stimulation of anti-CCR5 antibodies has, therefore, been shown to be an effective way to prevent HIV infection, at least in principal. Further studies will be needed to test whether the strategy outlined here will successfully break tolerance in an auto antigen setting, whether development of autoantibody is associated with any pathology, and whether immunisation efficiency can be increased so as to stimulate antibody in a large majority of the population at risk.
